# Genetic polymorphism of *WNT9A* is functionally associated with thumb osteoarthritis in the Chinese population

**DOI:** 10.1186/s42358-023-00337-9

**Published:** 2024-01-29

**Authors:** Jian Dai, Haitao Jiang, Zhang Cheng, Yao Li, Zhaoqi Yang, Chuan Cheng, Xiaoming Tang

**Affiliations:** 1 Department of Orthopedics Surgery, Huai’an First People’s Hospital, Nanjing Medical University, Huai’an, Jiangsu Province China; 2 Postgraduate in Orthopedics Surgery, Nanjing Medical University, Nanjing, Jiangsu Province China; 3 Department of Orthopedics Surgery, Third People’s Hospital of Jiujiang City, Jiujiang, Jiangxi Province China

**Keywords:** Thumb osteoarthritis, *WNT9A*, Variants, Etiology

## Abstract

**Background:**

In a recent genome-wide association study, novel genetic variations of *WNT9A* were reported to be involved in the etiopathogenesis of thumb osteoarthritis (TOA) in Caucasians. Our purposes were to replicate the association of *WNT9A* with the development of TOA in the Chinese population and to further unveil the functional role of the risk variants.

**Methods:**

SNP rs11588850 of *WNT9A* were genotyped in 953 TOA patients and 1124 healthy controls. The differences of genotype and allele distributions between the patients and healthy controls were evaluated using the Chi-square test. Luciferase Reporter Assay was performed to investigate the influence of variant on the gene expression.

**Results:**

There was significantly lower frequency of genotype AA in TOA patients than in the controls 74.9% vs. 81.9%, p < 0.001). The frequency of allele A was remarkably lower in the patients than in the controls (86.3% vs. 90.5%, p < 0.001), with an odds ratio of 0.66 (95% CI = 0.54–0.80). Luciferase Reporter Assay showed that the construct containing mutant allele G of rs11588850 displayed 29.1% higher enhancer activity than the wild allele A construct (p < 0.05).

**Conclusions:**

Allele G of rs11588850 was associated with the increased risk of TOA possibly via up-regulation of *WNT9A* expression. Further functional analysis into the regulatory role of rs11588850 in *WNT9A* expression can shed new light on the genetic architecture of TOA.

## Key Points

Genetic variants of *WNT9A* were associated with the incidence and severity of TOA.

Allele G of rs11588850 was associated with an increased transcriptional activity of *WNT9A* promoter.

Allele G of rs11588850 may add to the risk of TOA possibly via up-regulation of *WNT9A* expression.

Further functional analysis into the regulatory role of rs11588850 in *WNT9A* expression can shed new light on the genetic architecture of TOA.

## Introduction

Osteoarthritis (OA) is a chronic disease characterized by pain and decreased range of motion of the affected joints including knees, hips, hands and lower spine [1–3]. As a late-onset disease, the prevalence of OA increases with age [4, 5]. Hand OA (HOA) was reported to be a common type of OA, in addition to knee and hip OA. In the population aged more than 65 years, 38% of men and 62% of women had radiographic evidence of HOA [6–8]. People with HOA may have structural damage of hand joint accompanied by pain and stiffness, which eventually hamper their activities of daily living. It was reported by previous studies that HOA could confer severe clinical burden on the patients even comparable to rheumatoid arthritis [9, 10]. Although the exact aetiology of HOA remains largely unknown, systematic inflammation affecting articular cartilage, subchondral bone, synovial membrane, or ligaments has been reported as a potential risk factor [11, 12].

To date, numerous studies have been performed to unveil the genetic factors associated with hip or knee OA [13–15]. By contrast, there was a lack of knowledge concerning the etiopathogenesis of HOA. Therefore, identification or replication of novel variants associated with HOA is critical for a better understanding of the genetic background of this disease. Previous twin studies and familial clustering showed that HOA was a multifactorial disorder with strong genetic components [16]. The heritability of OA as estimated from twin studies ranged from 39 to 65% depending on the joint affected [17]. The risk of HOA in first-degree relatives of probands was two to five-fold higher than the general population [7]. To date, a number of candidate genes of HOA have been implicated by linkage and association studies. One genome-wide linkage analysis identified chromosome 2q11-q21 region as the susceptible locus for severe HOA in Finnish population [16]. Zhang et al. [18] reported one variant in *KLOTHO* gene is associated with the susceptibility of HOA through osteophyte formation in female Caucasian population. Wang et al. [19] reported that the minor allele of rs11177 was associated with increased susceptibility of HOA and clinical features of the patients in the Chinese population. However, these genes can explain only a small part of the genetic component.

Compared with candidate association study, genome-wide association study is a more powerful tool to uncover novel susceptible genes of human disease. Recently, Boer et al. [20] performed a genome-wide association study in 8700 HOA patients to determine novel associated genetic variations, which were further replicated in an independent cohort of 1203 patients. They identified a novel genetic locus for HOA on chromosome 1, and *WNT9A* was reported as a possible novel causal gene involved in the pathogenesis of thumb OA (TOA) in Caucasians [20]. To date, no replication study was reported to validate the association of *WNT9A* variant with the development of TOA in other populations. In this study, we recruited a cohort of TOA patients who received treatment in our clinic centers. Our purposes were to investigate the association of *WNT9A* with the development of TOA in the Chinese population and to further unveil the functional role of the risk variants.

## Methods

### Subjects

We retrospectively reviewed patients who visited our clinic centers due to swelling or pain of the hands between June 2016 and May 2020. Standard posteroanterior radiographs of both hands were taken for the patients, and joints of the thumb were assessed for radiographic TOA according to Kellgren/Lawrence (K/L) score as previously described [21]. The X-ray radiographs were independently evaluated by two senior surgeons. Radiographic changes of the joint were recorded for each patient, such as presence of osteophytes, joint-space narrowing, subchondral sclerosis, or cortical collapse. A joint was defined as OA affected if the K/L score was more than or equal to 2 [21]. Patients with two or more affected joints were recruited as TOA cases. Specifically, patients with nodal or erosive OA were all excluded from the study. The baseline characteristics of TOA patients were then collected from the medical records, including age, gender and body mass index (BMI). Pain analog scale (PAS) was used to evaluate the pain severity of the patients on a scale of 0–10, with 0 indicating no pain and 10 indicating the worst pain. Individuals with no affected joints of the hands were recruited as normal controls. All the controls were excluded to have TOA through X-rays of both hands. All the participants were excluded to have a history of gout, rheumatoid arthritis, hand joint surgery, or other chronic inflammatory diseases. All diagnostic procedures were performed in compliance with the Helsinki Declaration. Under the approval of the local ethics committee, written informed consents were obtained from the participants.

### Genotyping of target single nucleotide polymorphisms (SNP)s

Blood samples were collected from the subjects and the genomic DNA was extracted using the commercial kit (QIAGEN, Tokyo, Japan). Two SNPs of *WNT9A*, including rs10916199 and rs11588850, were genotyped with TaqMan SNP Genotyping Assay. The Amplification was performed in 30 µl reaction volumes, composed of 9 µl genomic DNA, 15 µl of the TaqMan Genotyping master mix, 3 µl TaqMan Genotyping assay mix, and 3 µl of distilled deionized water. The genotyping assay outcome was analyzed on ABI 7900HT Sequence Detection System (Applied Biosystem, Foster City, CA). 10% of the samples were randomly selected to validate the reproducibility of the genotyping outcome. 100% reproducibility was successfully confirmed.

### Cell cultures and luciferase reporter assay

The pGL3-basic plasmid (Bio Basic Inc. Markham, ON, Canada) was digested with SacI and MluI. Three constructs were synthesized and cloned into the pGL3-basic vector. The DNA fragment containing the promoter region of *WNT9A* was ligated to generate the pGL3-basic-P construct. Besides, the predicted enhancer sequences with different variants of rs11588850 (G or A allele) were ligated into the constructs, generating one constructs with mutant allele type (pGL3-basic-P-G) and the other construct with wide allele type (pGL3-basic-P-A). Renilla luciferase was used as an internal control, and pGL3-basic vector was used as a negative control. PureLink™ HiPure Plasmid Maxiprep Kit (Invitrogen, Waltham, MA, USA) was used to isolate the plasmid DNA.

Human embryonic kidney (HEK) 293T cells were cultured for the following Luciferase Reporter Assay, which were maintained at 37℃ and 5% CO2 in Dulbecco’s modified essential medium. Lipofectamine 2000 (Invitrogen, Waltham, MA, USA) was used to transfect the construct according to the manufacturer’s instructions. After transfection, the HEK293T cells were seeded into 96-well plates at a density of 10,000 cells per well for 24 h. The signal of luminescence was detected on an EnSpire™ Multilabel Plate Reader (Perkin Elmer, Waltham, MA, USA) with Firefly & Renilla Luciferase Single Tube Assay Kit (Biotium, Fremont, CA, USA). Three independent experiments with at least five technical replicates were performed for each construct. The relative luciferase activities were calculated with the activity of pGL3-basic construct defined as 1.

### Statistical analysis

The SPSS software (version 23.0, Chicago, USA) was used for statistical analysis. The continuous descriptive data were displayed as the mean ± standard deviation (SD). Inter-group comparison of the baseline characteristics was performed by the Student t test. For categorical data, the Chi-square test was used to compare the difference between the two groups. Hardy–Weinberg equilibrium tests were conducted in control samples to detect potential selection bias. The Chi-square analysis was used to compare the frequency of genotype and risk allele between the TOA cases and controls. The odds ratio (OR) and 95% confidential intervals (CIs) were calculated for each SNP. To analyze the relationship between risk variant and the clinical features, the Chi-square test was used to compare the distribution of genotypes in patients with different K/L scores (score 2, 3 or 4) or different PAS scores (1-3, 4-6, 7-10). A P-value of less than 0.05 was considered statistically significant. Genetic Association Study Power Calculator (https://csg.sph.umich.edu/abecasis/gas power calculator/) was used to estimate the statistical power of our sample size, which indicated more than 90% statistical power for detecting a SNP with an OR of more than 1.2.

## Results

### Baseline characteristics of the subjects

The demographic data of the patients were summarized in Table 1. There was no significant difference between the patients and the controls in terms of the mean age (47.8 ± 11.3 vs. 46.3 ± 13.4, p = 0.11), BMI (25.8 ± 5.3 vs. 25.5 ± 6.1, p = 0.24), smoking (p = 0.45), alcohol consumption (p = 0.32), or gender (p = 0.34). There were 314 (32.9%) patients scored as KL grades 2, 331 (34.7%) patients scored as KL grades 3, and 308 (32.3%) patients scored as KL grade 4, respectively. As for PAS score, 429 (45%) patients were rated as 1–3 (mild), 305 (32%) patients were rated as 4–6 (moderate) and 219 (23%) patients were rated as 7–10 (severe), respectively.

**Table 1:** Demographic data of the participants

	HOA Patients (n = 953)	Normal controls (n = 1124)	P
Age (years)	47.8 ± 11.3	46.9 ± 13.4	0.11
Gender			0.34
Male	511	627	
Female	442	497	
BMI (kg/m^2^)	25.8 ± 5.3	25.5 ± 6.1	0.24
Smoking			0.45
Yes	239	299	
No	714	825	
Alcohol consumption			0.32
Yes	317	398	
No	636	726	
KL grade, n (%)			
2	314 (32.9%)		
3	331 (34.7%)		
4	308 (32.3%)		
PAS, n (%)			
1–3	429 (45%)		
4–6	305 (32%)		
7–10	219 (23%)		

The continuous descriptive data were displayed as the mean ± standard deviation (SD)

### Replication of TOA-associated variants

HWE test showed no significant deviation regarding the distribution of the genotype frequency of rs10916199 and rs11588850 among the controls. As summarized in Tables 2, for rs10916199 of *WNT9A*, there was significantly lower frequency of genotype AA in TOA patients than in the controls (73.3% vs. 79.2%, p = 0.001). The frequency of allele A was remarkably lower in the patients than in the controls (85.4% vs. 88.9%, p = 0.001), with an odds ratio of 0.73 (95% CI = 0.61–0.88). Similarly, for rs11588850, there was significantly lower frequency of genotype AA in TOA patients than in the controls 74.9% vs. 81.9%, p < 0.001). The frequency of allele A was remarkably lower in the patients than in the controls (86.3% vs. 90.5%, p < 0.001), with an odds ratio of 0.66 (95% CI = 0.54–0.80). The two variants were in high LD (D’ = 0.93, r^2^ = 0.66).

**Table 2:** Comparison of the frequency of the genotype and allele for rs10916199 and rs11588850 between the patients and the controls

rs10916199 (A/G)		Genotype			p	Allele		p	Odds ratio (95% CI ^a^)
		AA	AG	GG		A	G		
	Patients ( n = 953)	699 (73.3%)	230 (24.1%)	24 (2.5%)	0.001	1628 (85.4%)	278 (14.6%)	0.001	0.73 (0.61–0.88)
	Controls ( n = 1124)	890 (79.2%)	219 (19.5%)	15 (1.3%)		1999 (88.9%)	249 (11.1%)		
rs11588850 (A/G)		Genotype			p	Allele		p	Odds ratio (95% CI ^a^)
		AA	AG	GG		A	G		
	Patients ( n = 953)	714 (74.9%)	217 (22.8%)	22 (2.3%)	< 0.001	1643 (86.3%)	261 (13.7%)	< 0.001	0.66 (0.54–0.80)
	Controls ( n = 1124)	920 (81.9%)	194 (17.2%)	10 (0.9%)		2034 (90.5%)	214 (9.5%)		

### Functional experiment of SNP rs11588850

**Fig. 1:**
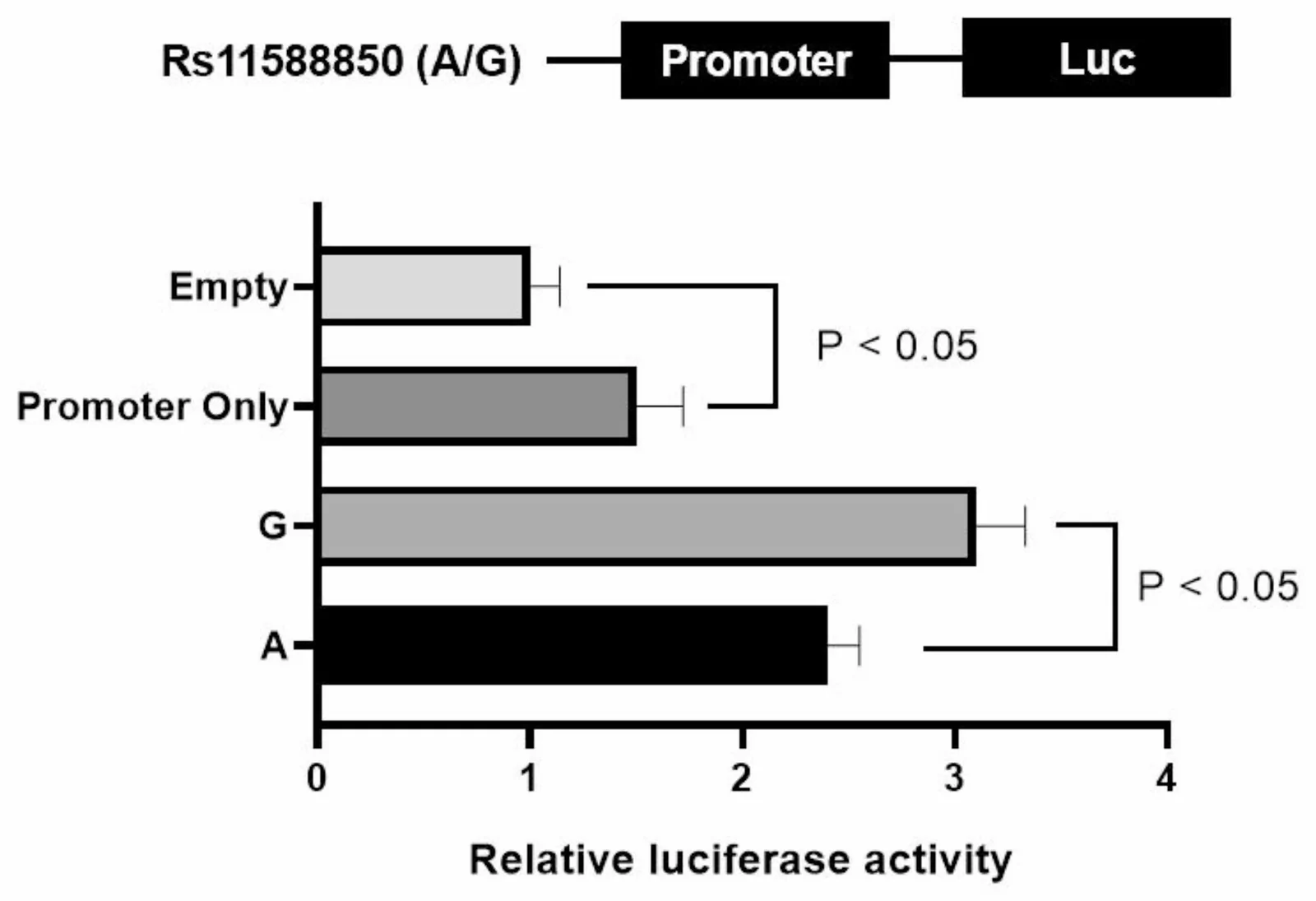
Outcome of Luciferase reporter assay. The Luciferase reporter assays for rs11588850 (A/G) on *WNT9A* promoter were performed in the HEK293 cell lines. Empty pGL3-basic vector was used as reference. The *WNT9A* promoter can remarkably increase the expression of reporter vector as compared to the empty pGL3-basic vector (p < 0.05). The mutant allele G construct presented 29.1% higher enhancer activity than the wild allele A construct (p < 0.05)

We performed a reporter assay to determine if variant rs11588850 could affect the regulation of gene expression. As shown in Fig. 1, the *WNT9A* promoter can remarkably increase the expression of reporter vector as compared to the empty pGL3-basic vector (p < 0.05). Moreover, the mutant allele G construct displayed 29.1% higher enhancer activity than the wild allele A construct (p < 0.05).

### Relationship between rs11588850 *and* clinical phenotypes of TOA

As shown in Table 3, rs11588850 was significantly associated with the severity of KL grade and PAS score in TOA patients. The frequency of genotype AA in patients with KL grade 2 was significantly higher than those with grade 3 or 4 (p = 0.01). Besides, the frequency of genotype AA in patients with PAS score of 1–3 was significantly higher than those with PAS score of 4–6 or 7–10 (p = 0.03).

**Table 3:** Relationship between rs11588850 and clinical features of the patients

Clinical features		Genotype of rs11588850		p
		AA (n = 714)	AG/GG (n = 239)	
KL grade	2 (n = 314)	256 (81.5%)	58 (18.5%)	0.01
	3 (n = 331)	246 (74.3%)	85 (25.7%)	
	4 (n = 308)	212 (68.8%)	96 (31.2%)	
PAS	1–3 (n = 429)	343 (79.9%)	86 (20.1%)	0.03
	4–6 (n = 305)	234 (76.7%)	71 (23.3%)	
	7–10 (n = 219)	147 (67.1%)	72 (32.9%)	

## Discussion

Previous GWAS has reported several novel variants in *WNT9A* which were associated with the risk of TOA in the European population [20]. To validate the role of *WNT9A* in the development of TOA, we replicated two novel variants of *WNT9A* and confirmed that rs10916199 and rs11588850 were significantly associated with TOA in the Chinese population. We found that both allele A of rs10916199 and allele A of rs11588850 could remarkably decrease the risk of TOA by 0.73 fold and 0.66 fold, respectively. This finding was consistent with the study of Boer et al. who reported a decreased TOA risk of 0.91 fold in subjects with allele A of rs10916199 from the European population. Difference regarding the OR of the risk allele between the Chinese population and the European population was noted, which we speculated could be attributed to the ethic difference. Herein, the association of variants in *WNT9A* with TOA was worthy of further replication in more populations on the basis of larger sample size.

Located in the promoter region of *WNT9A*, rs11588850 was predicted to affect the binding motif for *RAD21* which has been previously shown to bind to the *WNT9A* promoter region [20]. To investigate the molecular mechanism underlying the association of rs11588850 with TOA, for the first time, we analyzed the functional role of rs11588850 via the luciferase assay. Constructs containing the TOA -susceptibility allele (G allele) of rs11588850 showed remarkably higher enhancer activity than those containing the non-susceptibility allele A, indicating that the variant could affect the transcription level of *WNT9A*. In line with our findings, Boer et al. [20] reported that TOA patients had remarkably higher *WNT9A* expression in synovium tissues of knees than normal controls. Besides, the frequency of allele G of rs11588850 was significantly higher in TOA patients than in the normal controls. Taken together, it was plausible that allele G of rs11588850 was associated with the risk of TOA via up-regulation of *WNT9A* expression.

Previously known as *WNT14*, *WNT9A* is a member of the *WNT* gene family which has been reported to modulate key biological processes in development, growth, and homeostasis of the bone and joints [22]. Excessive activation of the *WNT* signaling pathway has been associated with the onset and severity of OA in the articular cartilage [23]. Hartmann et al. [24] reported that targeted misexpression of *WNT9A* led to down regulation of Sox9 and collagen type II in chondrocytes, concomitant with the histological appearance of a cartilaginous discontinuity. Knockout of *WNT9A* was reported to lead to a more severe disease phenotype in an arthritis model [25]. Interestingly, in this study, we observed that the risk allele of rs11588850 is associated with more severe pain and radiographic features of hand joint. Considering the role of rs11588850 in the regulation of *WNT9A* expression, it was probable that up-regulated *WNT9A* expression may indicate poor prognosis of TOA. The functional role of *WNT9A* in the progression of TOA was worthy of further investigation in the future study.

Several limitations of our study should be addressed here. First, we performed no in-vivo experiments to unveil the transcriptional factors that bind to the promoter region of *WNT9A*. More functional experiments such as electrophoretic mobility shift assay or chromatin immunoprecipitation assay are warranted to clarify the underlying regulatory mechanism. Second, the sample size of our study was relatively small, which might lead to selection bias of the cases. Third, more confounders such smoking, occupation and mechanical use of hand joint need to be included to evaluate the interaction between genetic factors and the environmental factors associated with TOA. Third, as the control group of our study were recruited through a free screening program of TOA in the community and most of the controls were male subjects. To match the control group, the case group in our study had more male patients than female patients, who were younger than 50 years predominantly. In the future study, the association between *WNT9A* and TOA can be validated in more female subjects aged more than 50 years.

## Conclusions

We validated that *WNT9A* was associated with the incidence and severity of TOA in the Chinese population. Allele G of rs11588850 was associated with the increased risk of TOA possibly via up-regulation of *WNT9A* expression. Further functional analysis into the regulatory role of rs11588850 in *WNT9A* expression can shed new light on the genetic architecture of TOA.

## Data Availability

All the data supporting our findings can be provided on request.
